# NALDB: nucleic acid ligand database for small molecules targeting nucleic acid

**DOI:** 10.1093/database/baw002

**Published:** 2016-02-19

**Authors:** Subodh Kumar Mishra, Amit Kumar

**Affiliations:** 1 Centre for Biosciences and Biomedical Engineering, Indian Institute of Technology Indore, Indore 452017, Madhya Pradesh, India

## Abstract

Nucleic acid ligand database (NALDB) is a unique database that provides detailed information about the experimental data of small molecules that were reported to target several types of nucleic acid structures. NALDB is the first ligand database that contains ligand information for all type of nucleic acid. NALDB contains more than 3500 ligand entries with detailed pharmacokinetic and pharmacodynamic information such as target name, target sequence, ligand 2D/3D structure, SMILES, molecular formula, molecular weight, net-formal charge, Alog*P*, number of rings, number of hydrogen bond donor and acceptor, potential energy along with their K_i_, K_d_, IC_50_ values. All these details at single platform would be helpful for the development and betterment of novel ligands targeting nucleic acids that could serve as a potential target in different diseases including cancers and neurological disorders. With maximum 255 conformers for each ligand entry, our database is a multi-conformer database and can facilitate the virtual screening process. NALDB provides powerful web-based search tools that make database searching efficient and simplified using option for text as well as for structure query. NALDB also provides multi-dimensional advanced search tool which can screen the database molecules on the basis of molecular properties of ligand provided by database users. A 3D structure visualization tool has also been included for 3D structure representation of ligands. NALDB offers an inclusive pharmacological information and the structurally flexible set of small molecules with their three-dimensional conformers that can accelerate the virtual screening and other modeling processes and eventually complement the nucleic acid-based drug discovery research. NALDB can be routinely updated and freely available on bsbe.iiti.ac.in/bsbe/naldb/HOME.php.

**Database URL:** http://bsbe.iiti.ac.in/bsbe/naldb/HOME.php

## Introduction

Nucleic acids are the key macromolecule elements in the cell and it regulates the cellular activities in several ways, at various stages such as replication, transcription, post-transcription, translation and also various processes such as host–pathogen interaction, affinity maturation of antibody, regulation of cellular stress, cell metabolism and many more (1–5). Cellular nucleic acids are majorly classified as DNA or RNA that may fold into different type of structures such as duplex, triplex, quadruplex, hairpin form, etc. (6–10). These structures have differences in their conformation as well as their stability such as RNA–RNA duplex shows the greater stability over the DNA–DNA, DNA–RNA or other hybrid duplex (11). Mutations either in the nucleic acid sequence or in their structure have adverse effects on the protein expression, its structure, localization or function that accounts for the diseased state in the cell. For instance, the formation of G-quadruplex structure inhibits the translation of RNA sequence found in 5′-UTR of *Arabidopsis thaliana* ATR mRNA (12). Similarly, the expression c9orf72 gene was inhibited due to the formation of G-quadruplex structure in the non-coding region of its mRNA and causes Amyotrophic lateral sclerosis (ALS), a neurodegenerative disease (13). Apart from inhibiting the gene expression, special structures of nucleic acid also have an important role in double-stranded break repair, for example, DSB-induced small RNA (14).

In addition, the triplet repeat RNA or DNA forms hairpin-like structures which have been found to be involved in several neurodegenerative disorders. The formation of these special structures could be regulated by binding of small molecules that stabilize or destabilize these structures. For example, binding of Porphyrin has been reported to distort G-quadruplex structure formed by 5′(r-GGGGCC)3′ repeat and could be used as a promising lead compound for the treatment of ALS and frontotemporal dementia (15). All these studies showed that nucleic acids are potential targets for majority of drugs used for the treatment of many diseases including cancer and various neurodegenerative disorders (15–17).

Since decades, vast nucleic acid-based targets were discovered and researchers are continuing to explore them, thereby providing a comprehensive set of parameters for their structure and functions. In addition, nucleic acids were also being targeted by small molecules as a potential therapeutics for various diseases. Small molecules either naturally available or synthetic ones were well-known binders of nucleic acid. These molecules bind to nucleic acid by various mechanisms such as chemical modification, cross linking, intercalation or by grooves binding (18–20). In general, small, cationic and planar molecules intercalates in between the base pairs while large non-planar molecule interacts by groove binding mechanism. Many of such molecules have been tested for their *in* *vitro* and *in* *vivo* activities and few of them have entered clinical trials. Thus, a huge experimental dataset containing various parameters for drug–nucleic acid interactions were being established. These experimental dataset contains useful information about various types of ligands and their detailed binding parameters for their interaction with the target nucleic acid. But, in literature all this information are scattered and it become difficult and challenging to gather complete set of information for a particular ligand and its targets. Future development and improvement in the drug discovery projects could be achieved by thorough understanding of these interactions. Therefore, it is requisite to compile all the experimental data and information at a single platform. Previously, many groups have provided various databases such as nucleic acid database (NDB), G4LDB, SMMRNA, etc. All these databases are dedicated to specific type of nucleic acid or its special structure. For instance, NDB contains all information about three-dimensional structure of nucleic acid and their complexes, G4LDB is dedicated to G-quadruplex ligands along with docking tool while SMMRNA describes about RNA structure, its complexes with small molecules (21, 22).

To the extent of our knowledge and information, there is no database that contains detailed experimental information for Drug–Nucleic acid binding such as K_d_, *T*_m_, IC_50_, etc. for all the majorly available type and forms of nucleic acid. Nucleic Acid Ligand Database (NALDB) is a unique portal that provides collective information for small molecules targeting various types of nucleic acid such as double-stranded DNA, double-stranded RNA, G-quadruplex DNA, G-quadruplex RNA, nucleic acid aptamers, triplex and hairpin or bulge containing DNA or RNA, on a single user interface. Enormous collection of ligands and detailed information about their targets, along with their mode of action for all type of nucleic acid structures makes this database idiosyncratic from other available databases. Our database is user friendly that allows user to screen a large set of molecules, their conformers as well as provides structure flexibility for ligands (structure). NALDB is a systematically organized database which contains most of the elementary information that are relevant for discovery and development of new therapeutics based on small molecule targeting nucleic acid. We believe that NALDB would potentially facilitate scientific community for their *in* *silico* lead identification and virtual screening process that speeds up the drug discovery process.

## Material and methods

### Database overview

The database is built using XAMPP which is a single package of Apache distribution containing web development and web testing tools such as MySQL, PHP and Perl. Apache (2.4.12 open SSL) is used as the web server platform and MySQL–RDMS (relational database management system) (5.6.24-MySQL Community Server) is used as database server for data storing, organization and query execution (see Supplementary File S1 for a complete description of MySQL database). Website pages were built using PHP language on Net-Beans IDE (8.0.2) platform. The NALDB site is best viewed by Google Chrome, Firefox and Opera browser enabled with Java (version 1.6 or higher).

### Data compendium

**Figure 1.**
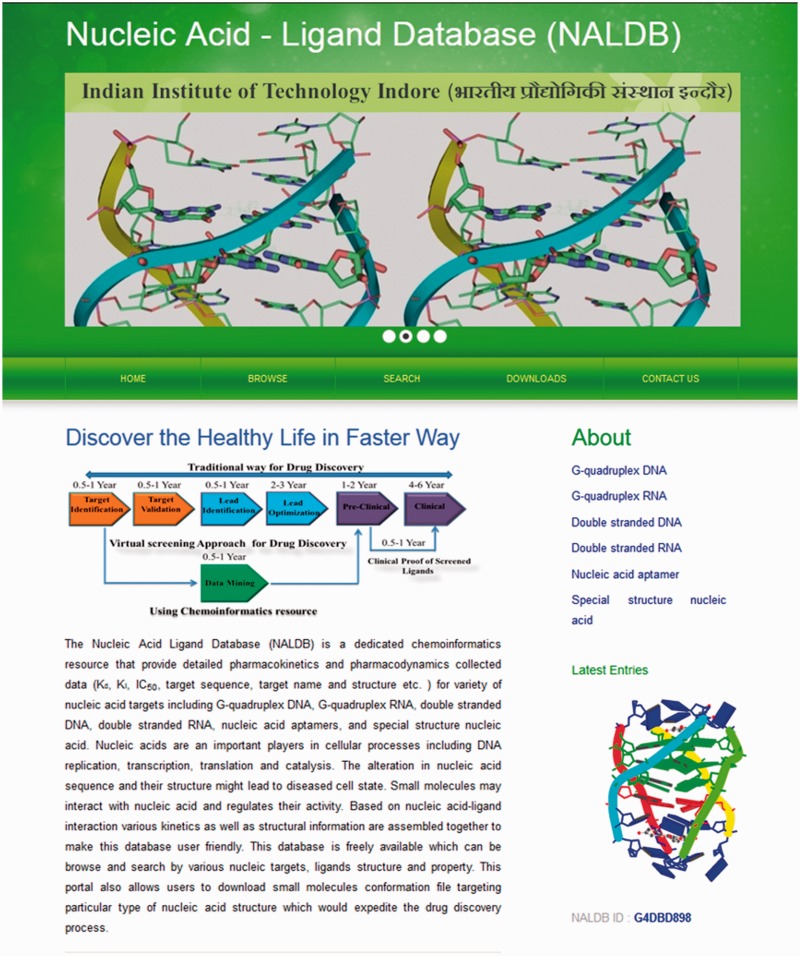
Home page of NALDB. Depicting the browse, search and download tools.

**Figure 2.**
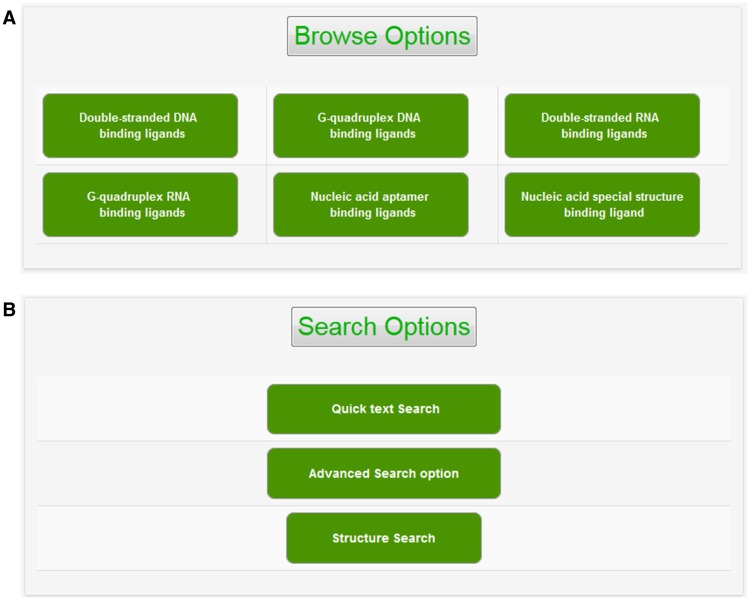
Browse and search options. Screenshot of (A) browse option showing classified organization of NALDB and (B) search options showing various search function.

The experimental data for target–ligand interaction were fetched from reported literature searched in PubMed and Google Scholar using various keywords such as ‘G-quadruplex’, ‘DNA binding drug’, ‘duplex or double-stranded DNA binding drug’, ‘RNA binding drug’ and ‘Aptamer binding drug’. The details of target sequence and its secondary structure, ligand structure, binding parameters for nucleic acid–ligand interactions like IC_50_ values, binding constants (K_d_), etc. were mined manually from each peer-reviewed research articles and reference PMIDs of each article were hyperlinked in the database table to provide the source of information for each ligand entry. The screenshot of NALDB home page showing search, browse and download option (Figures 1 and 2).

### Structures and molecular properties of ligands

**Figure 3.**
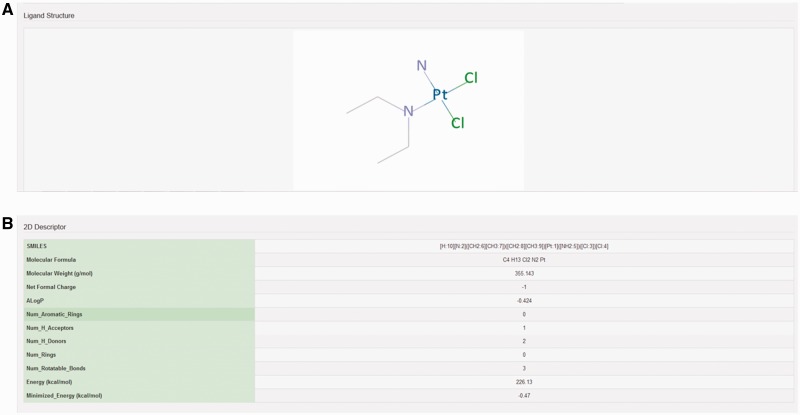
Structure and molecular properties of ligands. Screenshot showing example of (A) 2D structure of ligand and (B) 2D descriptors illustrating molecular properties of ligand.

The chemical structure of ligand structure and their abbreviated R groups were built using ACD/ChemSketch (Freeware) 2015 and were saved as ‘.mol file’ format. Their bad valences were fixed in Discovery studio 4.0 and 3D Coordinates were generated for each ligand. The geometry of each ligand was corrected and subsequently their 2D chemical structures were produced using Discovery Studio 4.0 (Accelerys Inc., San Diego, CA) (Figure 3A). The pharmacodynamic descriptors for each ligand such as molecular formula, molecular weight, formal charge, Alog*P*, number of aromatic rings, number of rings, number of H-bond donors, number of H-bond acceptors, number of rotatable bonds, minimized potential energy of the ligands were also calculated in Discovery Studio 4.0 (Figure 3B). The molecular formula of each ligand was displayed in PubChem format while molecular weight and minimized energy were displayed in g/mol and kcal/mol units, respectively.

### Structure search tool

**Figure 4.**
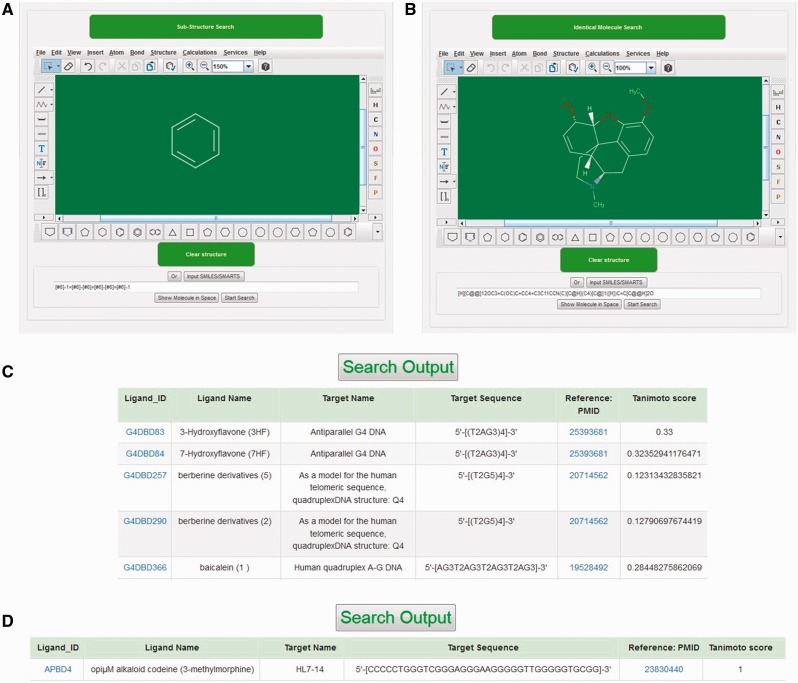
Structural search tool. Screenshot of (A) substructure search tool. (B) identical molecule search tool, (C) outputs of substructure search and (D) outputs of Identical molecule search.

Marvin Sketch 5.10.0 (ChemAxon) Java applet was used as a structure editor to facilitate structure search against reference molecules stored in NALDB database and could easily be run on Chrome, Firefox or Opera browser with suitable plugin (Java and Marvin version 1.6 or higher). This structure search tool could be used to either construct the query molecule on this Java applet or to import the query structure in .mol file format. When query structure was either drawn or imported in this structure search tool, the applet automatically converts the query structure into specific pattern that was considered as sub-graph. The search tool begins to match this sub-graph with graphs of reference molecules and the similarity between query structure and output ligand in the structure search result was quantified by tanimoto score. The tanimoto score is based on Jaccard similarity coefficient and the valid values for this score could be any of the possible float numbers between 0 and 1. There are two options available to search ligand structure: (i) substructure search, (ii) identical molecule search (Figure 4A and B). The substructure search function utilizes SMARTS line notation that meticulously specifies the chemical structure of ligand and gives an output with tanimoto score in the range of 0–1**.** This output has all those ligands from database that have molecular structure of queried molecule as a part of the complete ligand structure (Figure 4C). The identical molecule search function uses canonical SMILES to search the database molecules and generates an output of ligands that contains 100% similarity of structure with that of queried molecule structure with tanimoto score of 1 (Figure 4D). This applet also provides various types of ring templates and bond types to simplify the drawing of molecules containing multiple ring structure, bulky side chains with single, double, triple, aromatic, single up, single down, double *cis*, *trans* or coordinate bonds. The applet also facilitates the drawing of bigger molecule containing long carbon chains with chain templates. Several other inbuilt templates make this applet user friendly and allow the users to search structure with ease against the database molecule.

### Advanced search and quick search options

**Figure 5.**
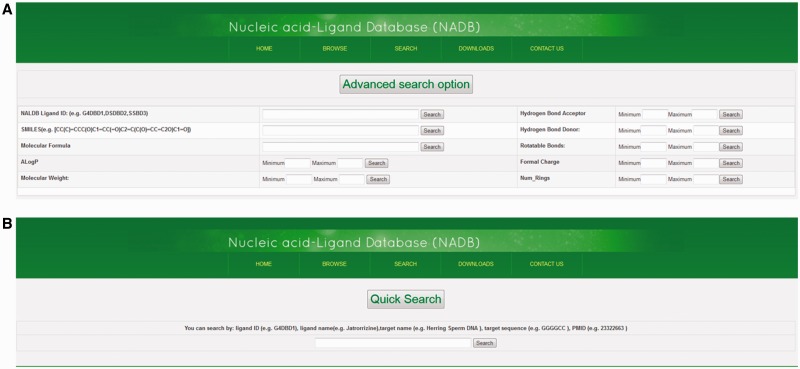
Advance search and quick search option. Screenshots showing (A) advance search function and (B) quick search function.

The advanced search option provides an efficient method to screen database molecules on the basis of their molecular properties. There are nine different types of molecular properties available for advanced search function, i.e. SMILES, Molecular Formula, ALog*P*, Molecular Weight, Hydrogen Bond Acceptor, Hydrogen Bond Donor, Rotatable Bonds, Formal Charge, and Numbers of Rings (see Supplementary File S2 for definition of descriptors). Users can select the range of molecular properties with their minimum and maximum values. Then the displayed data can be further sorted by molecular properties of ligand such as their molecular weight, molecular formula, IC_50_ value, ALog*P*, etc. Ligands can also be searched by using NALDB ligand IDs (Figure 5A). In the quick search option, users can search the database by various types of text queries such as ligand name, target sequence, target name or PMID. This provides additional search functionalities and helps the database users to quickly search the database by user friendly string search function (Figure 5B).

### Structure visualization of ligands

**Figure 6.**
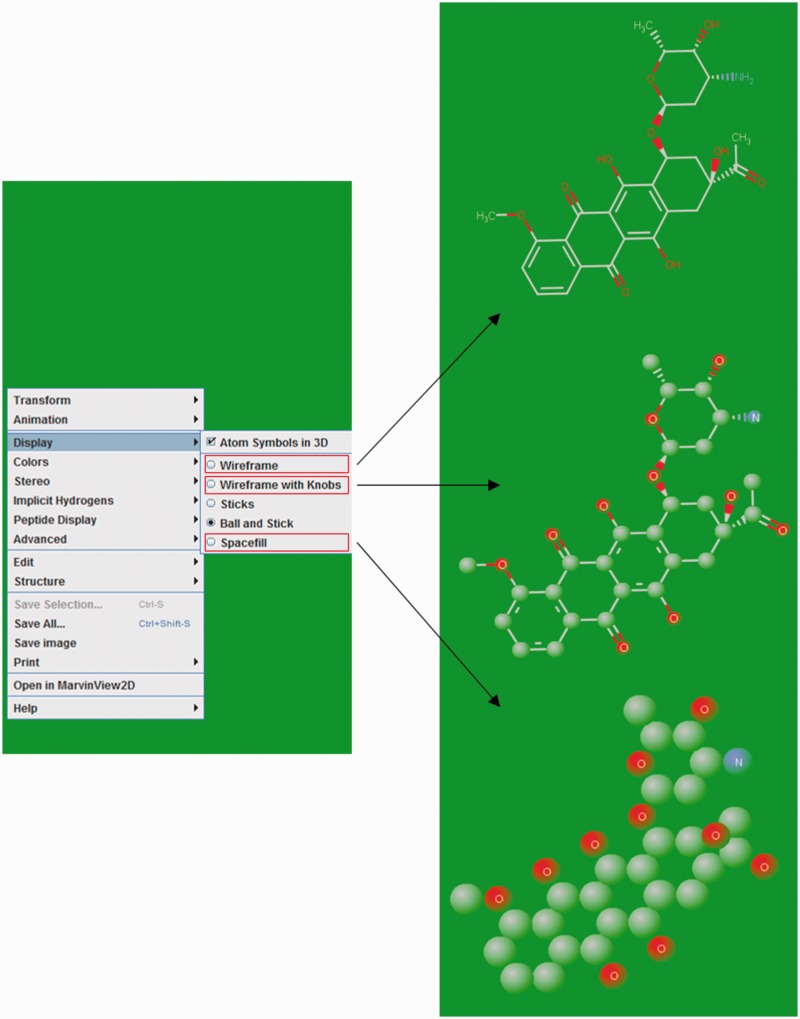
Ligand structure visualization. Screenshots of example showing various 3D structure representations.

The 2D structures of ligands were generated by Discovery Studio 4.0 and displayed as an image on their respective NALDB ligand ID page in the database webpage. MarvinView (ChemAxon) serves as molecular viewer for visualization of ligand’s 3D structures and displayed in a standard ball-and-stick model with CPK scheme as default color scheme. This default color scheme could be changed to either in shape or in group by right clicking in visualization panel. Ligands can also be displayed in other styles such as wireframe, wireframe with knobs and space fill model (Figure 6).

### 3D conformer generation

High-quality 3D conformers were created using ‘generate conformation’ protocol in Discovery Studio 4.0 and the best conformation method of this protocol was used. This method delivers better conformational coverage among all the available methods and conformers generated by this method could be further used for the 3D pharmacophore generation. These conformers were minimized using a SMART minimizer method by MMFF force-field with an energy threshold of 20 kcal/mol. A maximum of 255 conformations were generated for each molecule with RMS gradient of 0.1 (kcal/mol/Å). Conformer generation parameter file is described in Supplementary File S3.

## Results and discussion

### Browsing NALDB database

The graphical user interface (GUI) of NALDB database is available at the web URL http://bsbe.iiti.ac.in/bsbe/naldb/HOME.php. NALDB contains a total of 3610 entries that were broadly categorized into six browse options: (i) double-stranded DNA binding ligands, (ii) G-quadruplex DNA binding ligands, (iii) double-stranded RNA binding ligands, (iv) G-quadruplex RNA binding ligands, (v) nucleic acid aptamer binding ligands and (vi) nucleic acid special structure binding ligands. All these categories contain entries of ligands specific to each type of nucleic acid. These nucleic acid categories could be explored by clicking on them and that will open a new window containing list of ligands with their respective NALDB ID, target name, target sequence and reference (PMID). Users are allowed to sort the displayed data according to Drug_ID, target name, target sequence or their references. More details about chemo-informatics data of small molecules could be explored by further clicking the hyperlinked ligand ID that opens a new table listing binding details like IC_50_, K_d_, Δ*T*_m__,_ etc. 2D/3D structure and physiochemical properties such as number of hydrogen bond donors/acceptors, number of rings, SMILES, molecular weight, molecular formula etc. The 3D structure of ligands could be downloaded in the .mol file format. We have made the reference PMID as hyperlink to facilitate the users to retrieve the original source of experimental data such as molecular inhibition in cell lines, biophysical methods used to study ligands activities, etc. For example, ligand_ID G4DBD153, when browsed, resulting page will display the PMID as 24814531, hyperlinked with original PubMed link for the research article entitled as Polycyclic azoniahetarenes: assessing the binding parameters of complexes between unsubstituted ligands and G-quadruplex DNA. The GUI allows the users to perform text search, structure search, advanced search for both the target and ligands, it further allows downloading of the 3D conformers for the entire database molecules.

### Tools for structure similarity and search drug ability of ligands

We have embedded list of tools in the database to search database molecules on the basis of their structure and physiochemical properties. These tools are described in the following sections and this could be widely used for the drug development process.

### Advance search option

We have provided a search tool that filters the database molecules on the basis of drug likeness properties as defined by Lipinski’s Rule of Five (23). This rule describes the drug likeness of small molecule on the basis of their absorption that probably depends upon the number of hydrogen bond donors/acceptors, molecular and Alog*P* value. As per this rule, to have better drug ability for a molecule, the number of hydrogen bond acceptors should be > 10, number of hydrogen bond donors should be > 5, molecular weight of ligand should be > 500 and log*P* (partition coefficient) should be >5. Thus, the output molecules obtained from this advance search could facilitate further to undergo lead optimization processes as they were supposed have potential as drug candidate.

### Structure search tool

Structure-based search tool embedded in the database web page allows user to search database molecules that are similar or identical to the structure of query molecule. GUI of this search tool facilitates user either to draw ligand structure or to import ligands in .mol file format. Once the query structure was submitted, this search tool converts the query structure into their SMARTS descriptors which were subsequently transferred to database as a query string. This query string works as sub-graph used to search the graphs which are SMARTS descriptors of the reference molecules stored in database. This is the key step to perform the structure search. A graph represents a set of matrices that consists of a set of vertices (nodes) and group of edges (lines) representing the pattern of atoms and bonds in molecules, respectively. In structure search function, query structure and database molecules were treated as a pair for matching two structures. This matching is accomplished by graph matching protocol which is a mathematical construct that establishes the correlation between pairs. Here, in this case, query structure and database molecules were treated as a pair for matching two structures. The SMARTS descriptors of the reference molecule already stored in the database can be used as a graph to search against sub graph (string descriptors of query molecule). If search function finds a counterpart of query string (sub graph) from the database, it will generate a list of ligand containing query structure as part of complete ligand with their corresponding tanimoto score. For instance, when user provides hexagonal ring as query structure then substructure search function gives an output of all the ligands that have hexagonal ring structure in their complete structure along with their tanimoto score (Figure 4A). The tanimoto score displayed in result table describes the similarity between query and database molecule quantitatively. As tanimoto score suggests the similarity between query molecule and output molecules. Thus, the least value of tanimoto score corresponds to poor similarity between two structures while tanimoto score of 1 suggests 100% similar molecule.

### Quick search option

In order to search database rapidly rather than constructing the ligand structure, we have provided Quick search option in the database. This search option facilitates the user to search database directly by ligand or target name, target sequence, PMID, etc. For example, if user gives Daunomycin as query string, this search option gives the output that has Daunomycin as a small molecule. In another case, if user gives GGGGCC as an input query string, search result would list all the ligand that targets GGGGCC sequence.

### 3D conformation of database ligands

3D conformers are major requisite for performing high throughput screening such as virtual screening. Majorly, shape-based and pharmacophore-based screenings are the popular approaches and have been considered as valuable methods to perform the virtual screening (24–30). Shape-based virtual screening uses volume overlapping score while pharmacophore-based screening uses pharmacophore descriptors; such as groups containing H-bond donor and acceptor site, hydrophobic and electrostatic interaction sites, ring structure and other virtual points; of both the query and reference molecules (31). We have generated diverse ligand conformations for >2000 molecules in the database and categorized those in six different files according to nucleic acid category in the NALDB database. For each of the ligand available in database, we have generated a maximum of 255 conformers with the energy threshold of 20 kcal/mol. All these conformers could be easily download in .mol format which will help users to have better understanding of the various conformational state of the molecule, that could further be used as reference set of library for performing shape-based or pharmacophore-based virtual screening process.

## Conclusion and future prospects

We have built NALDB database with an attempt to assist the scientific community to improve further the efficacy of small molecule therapeutics for nucleic acid-based diseases. We have traversed >5000 peer-reviewed journals to gather the wide range of chemo-informatics data and compile them on a single platform. The NALDB is a collection of nucleic acid binding small molecule with >3500 entries. We have also provided multiple conformations of 3D structures for these molecules to facilitate the drug discovery process using shape-based and pharmacophore-based virtual screening. Users can browse our database on the basis of various categories of nucleic acid structures and have an ease to access several binding parameters as well as their activity. The conventional pharmacodynamics and pharmacokinetic information includes K_d_, K_i_, IC_50_ values, 3D and 2D structure, SMILES, molecular weight, hydrogen bond donors/acceptors and 3D conformation. The GUI of our database facilitate the users to search the database efficiently either by putting ligand name, target name, target sequence, PMID, molecular descriptors such as SMILES, molecular weight, number of hydrogen bond donor and acceptor, etc. We believe that NALDB would stand as chemically oriented portal for the advancement of structure-based drug design, virtual screening, molecular dynamic simulation and docking studies to develop the therapeutics for targeting nucleic acid-based diseases. We are continuing in process of growing this database with more entries for small molecule and add more experimental information such as techniques used to describe the ligand target interactions. In future version of NALDB, we would include the tool for clustering analysis, QSAR statistical analysis, 3D structure comparisons and web-based docking tool to determined ligand target interactions.

## Supplementary Data

Supplementary data are available at *Database* online.

Supplementary Data
