# Supplementary material for: NALDB: nucleic acid ligand database for small molecules targeting nucleic acid
Source: Database (Oxford). 2016 Feb 19;2016:baw002. doi: 10.1093/database/baw002 (PMC4761111; doi:10.1093/database/baw002)
Supplement: Supplementary Data [file supp_2016_baw002_index.html]

Supplementary Data 

# NALDB: nucleic acid ligand database for small molecules targeting nucleic acid

## Supplementary Data

files

- Supplementary Data - zip file
