# Supplementary material for: NALDB: nucleic acid ligand database for small molecules targeting nucleic acid
Source: Database (Oxford). 2016 Feb 19;2016:baw002. doi: 10.1093/database/baw002 (PMC4761111; doi:10.1093/database/baw002)
Supplement: Supplementary Data [file supp_baw002_suppl_data.zip › Supplementary File S1.doc]

**Supplementary File S1: NALDB Description**

**Database description:**

MySQL RDBMS (relational database management system) is used for storing data. The main tables are as follows:

1) dsdna (stores information about Target double stranded DNA)
2) g4dna (stores information about target G quadruplex DNA)
3) dsrna (stores information about Target double stranded RNA)
4) g4rna (stores information about Target G quadruplex RNA)
5) aptamer (stores information about Target aptamer)
6) spclstr (stores information about those target which have special structure RNA and DNA)
7) dsdrug (stores information about double stranded DNA binding ligand)
8) g4drug (stores information about G quadruplex DNA binding ligand)
9) dsrna_drug (stores information about double stranded RNA binding ligand)
10) g4rna_drug (stores information about G quadruplex RNA binding ligand)
11) aptamerdrug (stores information about aptamer binding ligand)
12) spclstrdrug (stores information about special structure DNA/RNA binding ligand)
13) spclstr_drug (stores information about target-ligand interaction)
14) g4rna_drug (stores information about target-ligand interaction)
15) g4dna_drug (stores information about target-ligand interaction)
16) dsrna_drug (stores information about target-ligand interaction)
17) dsdna_drug (stores information about target-ligand interaction)
18) aptamer_drug (stores information about target-ligand interaction)

Note: The following notations are used:

PRI: primary key, generally auto-generated by the database

UNI: this field will take only unique values

NULL: used to represent no value

varchar: MySQL datatype, used for storing a string of limited length efficiently

int: MySQL datatype, used to represent an integer

The Description of tables fields is given below:

1) **dsdna**

| Field | Type | Null | Collation | Default |
| --- | --- | --- | --- | --- |
| Drug_ID | varchar(500) | No | latin1_swedish_ci \| | None |
| Target_Name | varchar(500) | No | latin1_swedish_ci \| | -- |
| \| Target_Sequence | varchar(500) | Yes | latin1_swedish_ci \| | -- |
| Reference | varchar(500) | No | latin1_swedish_ci \| | N/A |

2) **g4dna**

| Field | Type | Null | Collation | Default |
| --- | --- | --- | --- | --- |
| Drug_ID | varchar(500) | No | latin1_swedish_ci \| | None |
| Target_Name | varchar(500) | No | latin1_swedish_ci \| | -- |
| \| Target_Sequence | varchar(500) | No | latin1_swedish_ci \| | -- |
| Reference | varchar(500) | No | latin1_swedish_ci \| | N/A |

3) dsrna

| Field | Type | Null | Collation | Default |
| --- | --- | --- | --- | --- |
| Drug_ID | varchar(500) | No | latin1_swedish_ci \| | None |
| Target_Name | varchar(500) | No | latin1_swedish_ci \| | -- |
| \| Target_Sequence | varchar(500) | Yes | latin1_swedish_ci \| | -- |
| Reference | varchar(500) | No | latin1_swedish_ci \| | N/A |

4) g4rna

| Field | Type | Null | Collation | Default |
| --- | --- | --- | --- | --- |
| Drug_ID | varchar(500) | No | latin1_swedish_ci \| | None |
| Target_Name | varchar(500) | No | latin1_swedish_ci \| | -- |
| \| Target_Sequence | varchar(500) | Yes | latin1_swedish_ci \| | -- |
| Reference | varchar(500) | No | latin1_swedish_ci \| | N/A |

5) aptamer

| Field | Type | Null | Collation | Default |
| --- | --- | --- | --- | --- |
| Drug_ID | varchar(500) | No | latin1_swedish_ci \| | None |
| Target_Name | varchar(500) | No | latin1_swedish_ci \| | -- |
| \| Target_Sequence | varchar(500) | Yes | latin1_swedish_ci \| | -- |
| Reference | varchar(500) | No | latin1_swedish_ci \| | N/A |

6) spclstr

| Field | Type | Null | Collation | Default |
| --- | --- | --- | --- | --- |
| Drug_ID | varchar(500) | No | latin1_swedish_ci \| | None |
| Target_Name | varchar(500) | No | latin1_swedish_ci \| | -- |
| \| Target_Sequence | varchar(500) | Yes | latin1_swedish_ci \| | -- |
| Reference | varchar(500) | No | latin1_swedish_ci \| | N/A |

7) dsdrug

| Field | Type | Null | Collation | Default |
| --- | --- | --- | --- | --- |
| Drug_ID | varchar(500) | No | latin1_swedish_ci \| | None |
| IUPAC_NAME | Text | No | latin1_swedish_ci \| | None |
| SMILES | Text | No | latin1_swedish_ci \| | None |
| Molecular_Formula | varchar(500) | No | latin1_swedish_ci \| | None |
| Molecular_Weight | varchar(500) | No | latin1_swedish_ci \| | None |
| NetFormalCharge | varchar(500) | No | latin1_swedish_ci \| | None |
| ALogP | varchar(500) | No | latin1_swedish_ci \| | None |
| Num_AromaticRings | varchar(500) | No | latin1_swedish_ci \| | None |
| Num_H_Acceptors | varchar(500) | No | latin1_swedish_ci \| | None |
| Num_H_Donors | varchar(500) | No | latin1_swedish_ci \| | None |
| Num_Rings | varchar(500) | No | latin1_swedish_ci \| | None |
| Num_RotatableBonds | varchar(500) | No | latin1_swedish_ci \| | None |
| Energy | varchar(500) | No | latin1_swedish_ci \| | None |
| Minimized_Eergy | varchar(500) | No | latin1_swedish_ci \| | None |

8) g4drug

| Field | Type | Null | Collation | Default |
| --- | --- | --- | --- | --- |
| Drug_ID | varchar(500) | No | latin1_swedish_ci \| | None |
| IUPAC_NAME | Text | No | latin1_swedish_ci \| | None |
| SMILES | Text | No | latin1_swedish_ci \| | None |
| Molecular_Formula | varchar(500) | No | latin1_swedish_ci \| | None |
| Molecular_Weight | varchar(500) | No | latin1_swedish_ci \| | None |
| NetFormalCharge | varchar(500) | No | latin1_swedish_ci \| | None |
| ALogP | varchar(500) | No | latin1_swedish_ci \| | None |
| Num_AromaticRings | varchar(500) | No | latin1_swedish_ci \| | None |
| Num_H_Acceptors | varchar(500) | No | latin1_swedish_ci \| | None |
| Num_H_Donors | varchar(500) | No | latin1_swedish_ci \| | None |
| Num_Rings | varchar(500) | No | latin1_swedish_ci \| | None |
| Num_RotatableBonds | varchar(500) | No | latin1_swedish_ci \| | None |
| Energy | varchar(500) | No | latin1_swedish_ci \| | None |
| Minimized_Eergy | varchar(500) | No | latin1_swedish_ci \| | None |

9) dsrna_drug

| Field | Type | Null | Collation | Default |
| --- | --- | --- | --- | --- |
| Drug_ID | varchar(500) | No | latin1_swedish_ci \| | None |
| IUPAC_NAME | Text | No | latin1_swedish_ci \| | None |
| SMILES | Text | No | latin1_swedish_ci \| | None |
| Molecular_Formula | varchar(500) | No | latin1_swedish_ci \| | None |
| Molecular_Weight | varchar(500) | No | latin1_swedish_ci \| | None |
| NetFormalCharge | varchar(500) | No | latin1_swedish_ci \| | None |
| ALogP | varchar(500) | No | latin1_swedish_ci \| | None |
| Num_AromaticRings | varchar(500) | No | latin1_swedish_ci \| | None |
| Num_H_Acceptors | varchar(500) | No | latin1_swedish_ci \| | None |
| Num_H_Donors | varchar(500) | No | latin1_swedish_ci \| | None |
| Num_Rings | varchar(500) | No | latin1_swedish_ci \| | None |
| Num_RotatableBonds | varchar(500) | No | latin1_swedish_ci \| | None |
| Energy | varchar(500) | No | latin1_swedish_ci \| | None |
| Minimized_Eergy | varchar(500) | No | latin1_swedish_ci \| | None |

10) g4rna_drug

| Field | Type | Null | Collation | Default |
| --- | --- | --- | --- | --- |
| Drug_ID | varchar(500) | No | latin1_swedish_ci \| | None |
| IUPAC_NAME | Text | No | latin1_swedish_ci \| | None |
| SMILES | Text | No | latin1_swedish_ci \| | None |
| Molecular_Formula | varchar(500) | No | latin1_swedish_ci \| | None |
| Molecular_Weight | varchar(500) | No | latin1_swedish_ci \| | None |
| NetFormalCharge | varchar(500) | No | latin1_swedish_ci \| | None |
| ALogP | varchar(500) | No | latin1_swedish_ci \| | None |
| Num_AromaticRings | varchar(500) | No | latin1_swedish_ci \| | None |
| Num_H_Acceptors | varchar(500) | No | latin1_swedish_ci \| | None |
| Num_H_Donors | varchar(500) | No | latin1_swedish_ci \| | None |
| Num_Rings | varchar(500) | No | latin1_swedish_ci \| | None |
| Num_RotatableBonds | varchar(500) | No | latin1_swedish_ci \| | None |
| Energy | varchar(500) | No | latin1_swedish_ci \| | None |
| Minimized_Eergy | varchar(500) | No | latin1_swedish_ci \| | None |

11) aptamerdrug

| Field | Type | Null | Collation | Default |
| --- | --- | --- | --- | --- |
| Drug_ID | varchar(500) | No | latin1_swedish_ci \| | None |
| IUPAC_NAME | Text | No | latin1_swedish_ci \| | None |
| SMILES | Text | No | latin1_swedish_ci \| | None |
| Molecular_Formula | varchar(500) | No | latin1_swedish_ci \| | None |
| Molecular_Weight | varchar(500) | No | latin1_swedish_ci \| | None |
| NetFormalCharge | varchar(500) | No | latin1_swedish_ci \| | None |
| ALogP | varchar(500) | No | latin1_swedish_ci \| | None |
| Num_AromaticRings | varchar(500) | No | latin1_swedish_ci \| | None |
| Num_H_Acceptors | varchar(500) | No | latin1_swedish_ci \| | None |
| Num_H_Donors | varchar(500) | No | latin1_swedish_ci \| | None |
| Num_Rings | varchar(500) | No | latin1_swedish_ci \| | None |
| Num_RotatableBonds | varchar(500) | No | latin1_swedish_ci \| | None |
| Energy | varchar(500) | No | latin1_swedish_ci \| | None |
| Minimized_Eergy | varchar(500) | No | latin1_swedish_ci \| | None |

12) spclstrdrug

| Field | Type | Null | Collation | Default |
| --- | --- | --- | --- | --- |
| Drug_ID | varchar(500) | No | latin1_swedish_ci \| | None |
| IUPAC_NAME | Text | No | latin1_swedish_ci \| | None |
| SMILES | Text | No | latin1_swedish_ci \| | None |
| Molecular_Formula | varchar(500) | No | latin1_swedish_ci \| | None |
| Molecular_Weight | varchar(500) | No | latin1_swedish_ci \| | None |
| NetFormalCharge | varchar(500) | No | latin1_swedish_ci \| | None |
| ALogP | varchar(500) | No | latin1_swedish_ci \| | None |
| Num_AromaticRings | varchar(500) | No | latin1_swedish_ci \| | None |
| Num_H_Acceptors | varchar(500) | No | latin1_swedish_ci \| | None |
| Num_H_Donors | varchar(500) | No | latin1_swedish_ci \| | None |
| Num_Rings | varchar(500) | No | latin1_swedish_ci \| | None |
| Num_RotatableBonds | varchar(500) | No | latin1_swedish_ci \| | None |
| Energy | varchar(500) | No | latin1_swedish_ci \| | None |
| Minimized_Eergy | varchar(500) | No | latin1_swedish_ci \| | None |

13) spclstr_drug

| Field | Type | Null | Collation | Default |
| --- | --- | --- | --- | --- |
| Drug_ID | longtext | No | utf8_unicode_ci | None |
| Target_Name | longtext | No | Utf8_unicode_ci | None |
| Target_Sequence | longtext | No | Utf8_unicode_ci | None |
| Liagnd_Name | longtext | No | Utf8_unicode_ci | None |
| Binding_detail_1 | longtext | No | Utf8_unicode_ci | None |
| Binding_detail_2 | longtext | No | Utf8_unicode_ci | None |
| Binding_detail_3 | longtext | No | Utf8_unicode_ci | None |
| Binding_detail_4 | longtext | No | Utf8_unicode_ci | None |
| Refference | longtext | No | Utf8_unicode_ci | None |

14) g4rna_drug

| Field | Type | Null | Collation | Default |
| --- | --- | --- | --- | --- |
| Drug_ID | longtext | No | utf8_unicode_ci | None |
| Target_Name | longtext | No | Utf8_unicode_ci | None |
| Target_Sequence | longtext | No | Utf8_unicode_ci | None |
| Liagnd_Name | longtext | No | Utf8_unicode_ci | None |
| Binding_detail_1 | longtext | No | Utf8_unicode_ci | None |
| Binding_detail_2 | longtext | No | Utf8_unicode_ci | None |
| Binding_detail_3 | longtext | No | Utf8_unicode_ci | None |
| Binding_detail_4 | longtext | No | Utf8_unicode_ci | None |
| Refference | longtext | No | Utf8_unicode_ci | None |

15) g4dna_drug

| Field | Type | Null | Collation | Default |
| --- | --- | --- | --- | --- |
| Drug_ID | longtext | No | utf8_unicode_ci | None |
| Target_Name | longtext | No | Utf8_unicode_ci | None |
| Target_Sequence | longtext | No | Utf8_unicode_ci | None |
| Liagnd_Name | longtext | No | Utf8_unicode_ci | None |
| Binding_detail_1 | longtext | No | Utf8_unicode_ci | None |
| Binding_detail_2 | longtext | No | Utf8_unicode_ci | None |
| Binding_detail_3 | longtext | No | Utf8_unicode_ci | None |
| Binding_detail_4 | longtext | No | Utf8_unicode_ci | None |
| Refference | longtext | No | Utf8_unicode_ci | None |

16) dsrna_drug

| Field | Type | Null | Collation | Default |
| --- | --- | --- | --- | --- |
| Drug_ID | longtext | No | utf8_unicode_ci | None |
| Target_Name | longtext | No | Utf8_unicode_ci | None |
| Target_Sequence | longtext | No | Utf8_unicode_ci | None |
| Liagnd_Name | longtext | No | Utf8_unicode_ci | None |
| Binding_detail_1 | longtext | No | Utf8_unicode_ci | None |
| Binding_detail_2 | longtext | No | Utf8_unicode_ci | None |
| Binding_detail_3 | longtext | No | Utf8_unicode_ci | None |
| Binding_detail_4 | longtext | No | Utf8_unicode_ci | None |
| Refference | longtext | No | Utf8_unicode_ci | None |

17) dsdna_drug

| Field | Type | Null | Collation | Default |
| --- | --- | --- | --- | --- |
| Drug_ID | longtext | No | utf8_unicode_ci | None |
| Target_Name | longtext | No | Utf8_unicode_ci | None |
| Target_Sequence | longtext | No | Utf8_unicode_ci | None |
| Liagnd_Name | longtext | No | Utf8_unicode_ci | None |
| Binding_detail_1 | longtext | No | Utf8_unicode_ci | None |
| Binding_detail_2 | longtext | No | Utf8_unicode_ci | None |
| Binding_detail_3 | longtext | No | Utf8_unicode_ci | None |
| Binding_detail_4 | longtext | No | Utf8_unicode_ci | None |
| Refference | longtext | No | Utf8_unicode_ci | None |

18) aptamer_drug

| Field | Type | Null | Collation | Default |
| --- | --- | --- | --- | --- |
| Drug_ID | longtext | No | utf8_unicode_ci | None |
| Target_Name | longtext | No | Utf8_unicode_ci | None |
| Target_Sequence | longtext | No | Utf8_unicode_ci | None |
| Liagnd_Name | longtext | No | Utf8_unicode_ci | None |
| Binding_detail_1 | longtext | No | Utf8_unicode_ci | None |
| Binding_detail_2 | longtext | No | Utf8_unicode_ci | None |
| Binding_detail_3 | longtext | No | Utf8_unicode_ci | None |
| Binding_detail_4 | longtext | No | Utf8_unicode_ci | None |
| Refference | longtext | No | Utf8_unicode_ci | None |
