# Supplementary material for: NALDB: nucleic acid ligand database for small molecules targeting nucleic acid
Source: Database (Oxford). 2016 Feb 19;2016:baw002. doi: 10.1093/database/baw002 (PMC4761111; doi:10.1093/database/baw002)
Supplement: Supplementary Data [file supp_baw002_suppl_data.zip › Supplementary File S2.doc]

**Supplementary File S2: Descriptors definition**

1. **SMILES**

SMILES (Simplified molecular-input line-entry system) are line notation which is used to describe the chemical species . In this notation atoms are described by their standard abbreviation such as [Fe] for iron, bonds are represented by single, double or triple line depend on the bond type, branches are represented with parenthesis. Configuration on the double bond are described by the “/” or “\” .

2. **ALogP**

This is used to represent the hydrophobicity of chemical species and measured as the ratio of concentration of compound in a mixture of two immiscible phases. AlogP calculation is based on the regression equation which is depend upon hydrophobicity contribution of 115 atom types. AlogP for any chemical species estimated by :

AlogP=∑_i_n_i_a_i_

Where n_i_ is number of atom type i and a_i_ corresponding hydrophobicity constant.

3. **Hydrogen Bond Acceptor**

This is a measure of the hydrogen-bonding ability of a molecule expressed in terms of number of possible hydrogen-bond acceptors. Specifically, it is calculated by adding up any nitrogen, oxygen and fluorine, excluding N with positive formal charge, higher oxidation states and pyrrolyl form of nitrogen.

4. **Hydrogen Bond Donor**

This is a measure of the hydrogen-bonding ability of a molecule expressed in terms of number of possible hydrogen-bond donors. Specifically, it is calculated by adding up the hydrogens bonded to any nitrogen and oxygen without negative charge in the molecule.

5. **Rotatable Bonds**

This is the total number of bonds in a ligand or small molecule which allow free rotation around themselves. These are defined as any single bond, not in a ring, bound to a nonterminal heavy atom. C-N bonds are excluded from this count because of their high rotational energy barrier.

6. **Formal Charge**

The formal charge of any chemical species is the charge assign to atom in that molecule, considering the sharing of chemical bond regardless of their electronegativity.
