# Supplementary material for: NALDB: nucleic acid ligand database for small molecules targeting nucleic acid
Source: Database (Oxford). 2016 Feb 19;2016:baw002. doi: 10.1093/database/baw002 (PMC4761111; doi:10.1093/database/baw002)
Supplement: Supplementary Data [file supp_baw002_suppl_data.zip › Supplementary File S3.doc]

**Supplementary File S3. The parameter used for generating 3D conformations of NALDB ligands.**

| **Conformation Method** | BEST |
| --- | --- |
| **Maximum Conformations** | 255 |
| **Energy Threshold** | 20.0 |
| **Discard Existing Conformations** | False |
| **Separate Conformations** | False |
| **Input Forcefield** | CHARMm |
| **Maximum Systematic Conformations** | 1000 |
| **Boltzmann Temperature** | 300 |
| **RMSD Cutoff** | 0.2 |
| **Torsion Increment** | Default |
| **Sp3-Sp3** | 120 |
| **Sp3-Sp2** | 120 |
| **Sp2-Sp2** | 180 |
| **Conformation Minimization** | True |
| **Minimization Algorithm** | Smart Minimizer |
| **Minimization Max Steps** | 2000 |
| **Minimization RMS Gradient** | 0.1 |
| **Minimization Forcefield** | CHARMm |
| **Minimization Partial Charge Estimation** | Momany-Rone |
| **Minimization Dielectric Constant** | 1 |
| **Implicit Solvent Dielectric Constant** | 80 |
